# Characterization of the complete chloroplast genome of *Calophaca sinica* Rehd.

**DOI:** 10.1080/23802359.2019.1710608

**Published:** 2020-01-14

**Authors:** Bin Guo, Zhi-Li Zheng, Kai Gao, Jia-Lin Ma, Jian-Yi Wang, Fei Yang, Xin-Min An

**Affiliations:** a National Engineering Laboratory for Tree Breeding, Beijing Forestry University, Beijing, People’s Republic of China;; b Shanxi Academy of Forest Sciences, Taiyuan, People’s Republic of China

**Keywords:** *Calophaca sinica*, chloroplast genome, phylogenetic analysis

## Abstract

*Calophaca sinica* Rehd. is a tree species with high economic value, whose resource has been declining due to unreasonable exploitation. In this study, we sequenced, assembled, and annotated the complete chloroplast genome of *C. sinica*. The whole chloroplast genome size is 129,345 bp, it lacks an inverted repeat (IR) region. The GC content of the whole chloroplast genome is 34.51%. The chloroplast genome comprises 112 unique genes, including 77 protein-coding genes (PCGs), 30 transfer RNA (tRNA) genes, and 5 ribosomal RNA (rRNA) genes. Phylogenetic analyses of chloroplast genomes derived from 15 species indicated that *C. sinica* is close to *Caragana* and *Tibetia* species in Papilionoideae.

The genus *Calophaca* Fisch. is composed of eight species, most of which are distributed in Central Asia. However, *Calophaca sinica* is found in the mountains of North China in East Asia, which makes the extension of *Calophaca* distribution area further toward the east and confers important phytogeographic significance (Zhang et al. 2015). In this study, we reported the complete sequence of the chloroplast genome of *C. sinica*, which will lay important groundwork for further studies on phylogenetic analysis and conservation genetics of this Endangered species. The annotated chloroplast genome has been deposited into GenBank with the accession number: MN696543.

Fresh *C. sinica* leaves were collected from Taiyuan of Shanxi Province, China (37°43′57″N, 112°28′22″E). The voucher specimens were deposited in Shanxi Academy of Forest Sciences (accession number: 20190820CS01). The total genomic DNA was extracted using the DNAsecure Plant Kit and sequenced by Illumina Hiseq2500 System. The obtained reads were assembled using the CANU *de nove* assembler (Koren et al. 2017). Complete chloroplast genome was annotated with the CpGAVAS (Liu et al. 2012) and DOGMA (Wyman et al. 2004).

The size of the chloroplast genome in *C. sinica* is 129,345 bp and lacks an inverted repeat (IR) region. The whole chloroplast genome encodes a total of 112 genes with known functions, which are identified and classified into 77 protein-coding genes, 30 tRNA genes, and 5 rRNA genes. The GC content represented 34.51% of the whole-genome. Most of the genes are single copy, whereas two genes present in double copies, 1 tRNA genes (*trnN-GUU*), and 1 rRNA genes (*rrn16*). 10 protein-coding genes (*rpoC1*, *atpF*, *clpP*, *petB*, *petD*, *rpl16*, *rpl2*, *ndhB*, *rps12*, *ndhA*) and five tRNA genes (*trnK-UUU*, *trnV-UAC*, *trnL-CAA*, *trnG-UCC*, *trnA-UGC*) are found to possess a single intron, whereas 1 gene (*ycf3*) contains two introns.

To determine the phylogenetic relationships among Papilionoideae, we downloaded the chloroplast genomes of 14 plant species belonging to Papilionoideae and one plant species of Cercidoideae (outgroup) from GenBank and constructed ML trees. Complete chloroplast genome sequence was aligned using the MAFFT v. 7 software and manually edited where required (Katoh and Standley 2013). The ML method was performed using 1000 bootstrap replicates in the IQ-TREE software (Nguyen et al. 2015). The result showed *C. sinica* is close with *Caragana* and *Tibetia* species in Papilionoideae (Figure 1). The complete chloroplast genome sequence of *C. sinica* will provide a useful resource for the conservation genetics of this species as well as for the phylogenetic studies of Papilionoideae.

**Figure 1.**
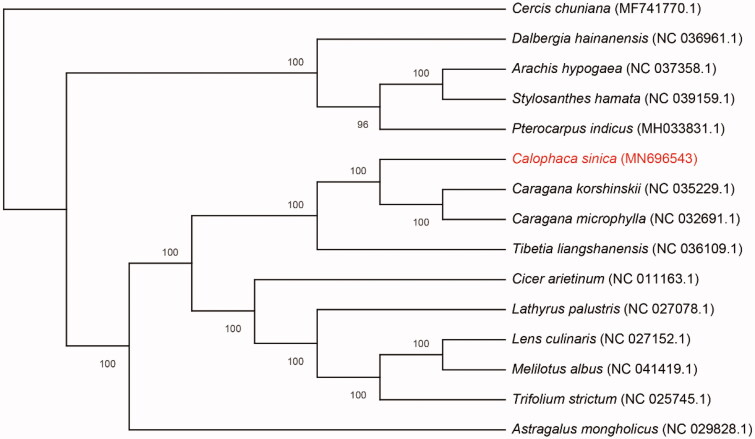
Maximum likelihood phylogenetic tree based on the complete chloroplast genome sequences of 15 plant species, *Cercis chuniana* was used as an outgroup. Numbers on the nodes are bootstrap values with 1000 replicates and bootstrap values of 100 were omitted.
